# A rapid and simple method for constructing stable mutants of *Acinetobacter baumannii*

**DOI:** 10.1186/1471-2180-10-279

**Published:** 2010-11-09

**Authors:** Jesús Aranda, Margarita Poza, Belén G Pardo, Soraya Rumbo, Carlos Rumbo, José R Parreira, Patricia Rodríguez-Velo, Germán Bou

**Affiliations:** 1 Servizo de Microbioloxía-INIBIC, Complexo Hospitalario Universitario A Coruña, As Xubias s/n, 15006. A Coruña, Spain; 2 Departamento de Xenética. Facultade de Veterinaria. Universidade de Santiago de Compostela. Campus de Lugo. 27002 Lugo, Spain

## Abstract

**Background:**

*Acinetobacter baumannii *is a multidrug-resistant bacterium responsible for nosocomial infections in hospitals worldwide. Study of mutant phenotypes is fundamental for understanding gene function. The methodologies developed to inactivate *A. baumannii *genes are complicated and time-consuming; sometimes result in unstable mutants, and do not enable construction of double (or more) gene knockout mutant strains of *A. baumannii*.

**Results:**

We describe here a rapid and simple method of obtaining *A. baumannii *mutants by gene replacement via double crossover recombination, by use of a PCR product that carries an antibiotic resistance cassette flanked by regions homologous to the target locus. To demonstrate the reproducibility of the approach, we produced mutants of three different chromosomal genes (*omp33*, *oxyR*, and *soxR*) by this method. In addition, we disrupted one of these genes (*omp33*) by integration of a plasmid into the chromosome by single crossover recombination, the most widely used method of obtaining *A. baumannii *mutants. Comparison of the different techniques revealed absolute stability when the gene was replaced by a double recombination event, whereas up to 40% of the population reverted to wild-type when the plasmid was disrupting the target gene after 10 passages in broth without selective pressure. Moreover, we demonstrate that the combination of both gene disruption and gene replacement techniques is an easy and useful procedure for obtaining double gene knockout mutants in *A. baumannii*.

**Conclusions:**

This study provides a rapid and simple method of obtaining stable mutants of *A. baumannii *free of foreign plasmidic DNA, which does not require cloning steps, and enables construction of multiple gene knockout mutants.

## Background

*Acinetobacter baumannii *is a Gram-negative coccobacillus that is increasingly recognized as a major pathogen causing nosocomial infections worldwide, particularly in patients admitted to intensive care units [1,2]. *A. baumannii *can cause pneumonia, wound infections, urinary tract infections, bacteremia and meningitis [3,4]. Its clinical significance, especially in recent years, has increased because of the ability of the bacterium to acquire resistance determinants, making it one of the microorganisms threatening the current antibiotic era [5].

The availability of the genome sequences of several strains of *A. baumannii *opens up new perspectives in the study of this bacterial species [6-9]. The artificial introduction of mutations, by molecular techniques, is a useful way of advancing our understanding of the genetics of *A. baumannii*. The method most commonly used to generate *A. baumannii *mutants involves integration of a plasmid into the chromosome by single crossover recombination. This method requires an internal fragment homologous to the target gene cloned into a suicide vector carrying resistance cassettes [10], which is a major limitation for systematic construction of mutants in post-genomic studies of *A. baumannii*. The possibility that a second crossover event will return the mutant to a wild-type phenotype is another important inconvenience. The gene replacement method is a useful way of overcoming these limitations.

Gene replacement typically involves transformation of a non-replicating plasmid containing a deleted or modified gene, followed by low-frequency integration of a plasmid into the chromosome and selection for resolution events to identify gene replacement candidates. In fact, in *A. baumannii*, the plasmids pSSK10, pEX100T, and pJQ200 are valuable tools for constructing mutants by this methodology [11-13]. However, these gene replacement methodologies require several subcloning steps and phenotypic screenings. As a means of circumventing these complicated approaches, we have developed a rapid and simple method of inactivating of chromosomal genes that does not require cloning steps. Moreover, the mutants grow directly on agar plates containing appropriate antibiotics and are confirmed by a simple PCR assay.

Integration of a linear piece of foreign DNA requires two recombination events, whereby the original genetic material is replaced by the recombinant DNA [14]. The methodology used in the present study is based on electroporation of a recipient *A. baumannii *strain with a linear PCR fragment carrying an antibiotic resistance cassette flanked by regions homologous to the target locus. This method was used successfully to inactivate three chromosomal loci in *A. baumannii *(*omp33*, *oxyR*, and *soxR*). Moreover, the stability of the mutant, gene expression, and the efficiency of gene disruption and gene replacement methods were compared. Finally, the combination of both techniques was found to be an easy and useful method of obtaining double knockout mutants of *A. baumannii*.

## Results

### Replacement of the *A. baumannii omp33 *gene

**Figure 1:**
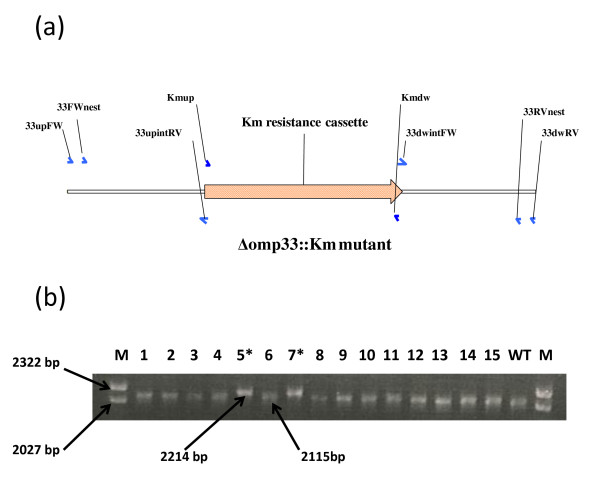
***omp33 *replacement**. (a) Schematic representation of the linear DNA constructed for the *omp33 *gene replacement, which was completely deleted. The oligonucleotides used (small arrows) are listed in Table 2. (b) Screening of *omp33 A. baumannii *mutants generated by gene replacement. The numbers at the top are bacterial colony numbers. WT, Wild-type control with 2115 bp. Colonies 5 and 7 (lanes 5* and 7*) with 2214 bp (2115 bp - 834 bp [from *omp33 *deletion] + 933 bp [from kanamycin insertion]) were sequenced to confirm gene replacement. Lambda DNA-*Hin*d III and ϕX174 DNA-*Hae *III Mix (Finnzymes) was used as a size marker (M). The lengths of PCR products and of some molecular size marker fragments are also indicated.

A PCR product containing a kanamycin resistance cassette flanked by 500 bp of the regions surrounding the *omp33 *gene (Figure 1a, Table 1) was introduced into the *A. baumannii *ATCC 17978 strain by electroporation. After selection on kanamycin-containing plates, the *A. baumannii *Δomp33::Km mutant was obtained. The frequency of generation of mutants by gene replacement was approximately 10^-7^. The PCR tests with locus-specific primers revealed that 2 of 15 clones obtained had replaced the wild-type gene by the kanamycin cassette (Figure 1b). In addition, allelic replacement in mutant clones was further confirmed by sequencing the PCR products obtained (data not shown).

**Table 1:** Genes of *A. baumannii *strain ATCC 17978 inactivated in the present study.

Product Name	Gene location^a^	Length^b^	Locus tag^c^	Accession number
Outer membrane protein (Omp33)	3789880 to 3790566	228	A1S_3297	YP_001086288.1
Transcriptional regulator SoxR	1547914 to 1548219	101	A1S_1320	YP_001084350.1
Transcriptional regulator OxyR	1150365 to 1151153	262	A1S_0992	YP_001084026.1

**^a^***A. baumannii *ATCC 17978 chromosomal coordinates for each gene.

**^b^**The length is expressed as number of amino acids.

**^c^**Based on National Center for Biotechnology Information http://www.ncbi.nlm.nih.gov

### Disruption of the *A. baumannii omp33 *gene

**Figure 2:**
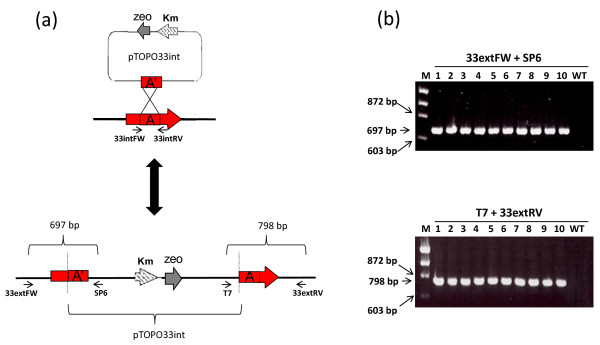
***omp33 *disruption**. (a) Schematic representation of the strategy used to construct the *omp33 *mutant by gene disruption (omp33::TOPO). The oligonucleotides used (small arrows) are listed in Table 2. The boxes indicated by A and A' represent the original and the cloned internal fragment of the *omp33 *gene, respectively. See Materials and Methods for details. (b) Screening of *omp33 A. baumannii *mutants generated by gene disruption. The numbers at the top are bacterial colony numbers. All PCR products with 697 bp and 798 bp (amplified with primer pairs 33extFW + SP6 and T7 + 33extRV, respectively) were sequenced to confirm *omp33 *gene disruption. Lambda DNA-*Hin*d III and ϕX174 DNA-*Hae *III Mix (Finnzymes) was used as a size marker (M). The wild-type strain (WT) was used as a negative control. The lengths of PCR products and of some molecular size marker fragments are also indicated.

The gene disruption method was also used to inactivate the *omp33 *gene. Gene disruption was carried out by cloning a 387-pb internal fragment of the *omp33 *gene into the pCR-BluntII-TOPO, to obtain the pTOPO33int plasmid (Figure 2a). After transformation of the recombinant plasmid into the *A. baumannii *ATCC 17978 strain and selection on kanamycin-containing plates, the *A. baumannii *omp33::TOPO mutant was obtained. The frequency of generation of mutants by gene disruption was approximately 10^-5^. PCR tests with locus-specific primers revealed that all the clones analyzed (10 of approximately 100) contained fragments of the expected size (Figure 2b). In addition, gene disruption in mutant clones was further confirmed by sequencing the PCR products obtained (data not shown).

### Stable maintenance of plasmid insertion into the chromosome requires drugselection

Gene knockout stability was tested by culturing both the Δomp33::Km and omp33::TOPO *A. baumannii *mutants under nonselective conditions (in the absence of antibiotics). Cultures of the mutant strains were initially grown in LB and at passages 1, 5, and 10, the cultures were dilution plated to obtain individual colonies, with replicate platings of 100 colonies for each strain on LB and LB supplemented with kanamycin. The frequency of loss of kanamycin resistance in each passage after growth in non-selective conditions was 1% (first), 9% (fifth), and 37% (tenth) for the gene disrupted omp33::TOPO mutant. By contrast, the gene-replaced Δomp33::Km mutant was stable since no reversions were detected in any passage. As expected, when the same experiment was carried out in the presence of selective pressure, both mutants remained stable (all colonies analyzed were resistant to kanamycin).

### Complementation

**Figure 3:**
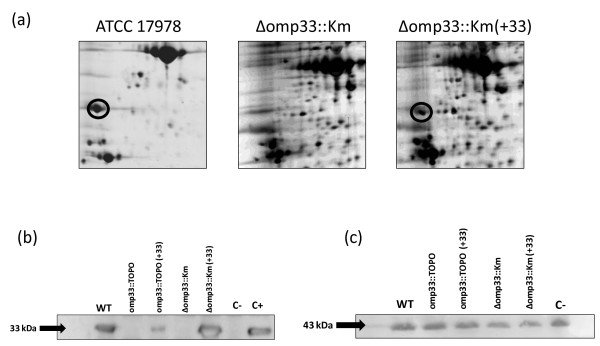
**Omp33 detection**. (a) 2-DE gels showing *A. baumannii *proteins from the wild-type strain (ATCC 17978), Δomp33::Km mutant, and Δomp33::Km mutant complemented with pET-RA-OMP33 plasmid (+33). The black circles indicate the Omp33 protein. (b) Western blot analysis showing the detection of the Omp33 protein in the protein extracts obtained from the wild-type and the pETRA-OMP33- complemented mutant strains. (+33): Strains complemented with the pETRA-OMP33 plasmid. C-: Δomp33::Km mutant containing the pET-RA vector (without the *omp33 *gene) as a negative control. The last lane (C+) indicates detection of the purified Omp33 protein used as a positive control. (c) Reversible staining of the membrane containing the transferred protein extracts from the indicated strains showing similar amounts of the majority protein (43 kDa) prior to Western blot analysis.

Taking advantage of the fact that the Omp33 protein has been identified in the proteome of *A. baumannii *ATCC 17978 strain by 2-DE and MALDITOF/TOF [15], we observed the absence of the Omp33 protein by 2-DE analysis of the Δomp33::Km mutant (Figure 3a). In order to complement the mutant phenotype, we constructed and tested the expression plasmid pET-RA. The wild-type *omp33 *gene without its promoter region was cloned into this expression plasmid. This construction was then introduced into the Δomp33::Km mutant strain by electroporation. The cell surface-associated proteins of the wild-type strain and the Δomp33::Km mutant strain complemented with the pET-RA-OMP33 plasmid were extracted and analyzed by 2DE. The Omp33 protein was detected in the mutant complemented with the Omp33 ORF under the control of the β-lactamase CTX-M14 gene promoter of the pET-RA plasmid (Figure 3a).

### Omp33 detection

Western blot analysis was performed for further confirmation of the absence of Omp33 in the *A. baumannii *mutants. For this purpose, cell surface-associated proteins of wild-type strain, *omp33 *mutants, and pET-RA-OMP33-complemented mutants were extracted and subjected to Omp33 Western blot analysis (Figure 3b). The Omp33 protein was not detected in the cell surface-associated proteins of the mutants. As expected, the Omp33 protein was detected in the cell surface-associated proteins of both Δomp33::Km and omp33::TOPO mutants containing the pET-RAOMP33 vector.

### Reproducibility of the gene replacement method

**Figure 4:**
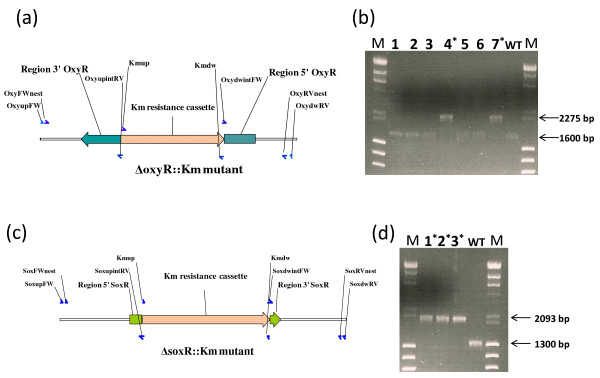
***oxyR *and *soxR *replacement**. (a) Schematic representation of the linear DNA constructed for the *oxyR *gene replacement. The oligonucleotides used (small arrows) are listed in Table 2. (b) Screening of *oxyR A. baumannii *mutants generated by gene replacement. The numbers at the top are bacterial colony numbers. WT; Wild-type control showing 1600 bp. Colonies 4 and 7 (lanes 4* and 7*) showing 2275 bp (1600 pb - 258 bp [from *oxyR *deletion] + 933 bp [from kanamycin insertion]) were sequenced to confirm gene replacement. Lambda DNA-*Hin*d III and ϕX174 DNA-*Hae *III Mix (Finnzymes) was used as a size marker (M). (c) Schematic representation of the linear DNA constructed for the *soxR *gene replacement. The oligonucleotides used (small arrows) are listed in Table 2. (d) Screening of *soxR A. baumannii *mutants generated by gene replacement. WT: Wild-type control with 1300 bp. Colonies 1, 2, and 3 (lanes 1*, 2*, and 3*) with 2093 bp (1300 bp - 140 bp [from *soxR *deletion] + 933 bp [from kanamycin insertion]) were sequenced to confirm gene replacement. Lambda DNA-*Hin*d III and ϕX174 DNA-*Hae *III Mix (Finnzymes) was used as a size marker (M).

**Figure 5:**
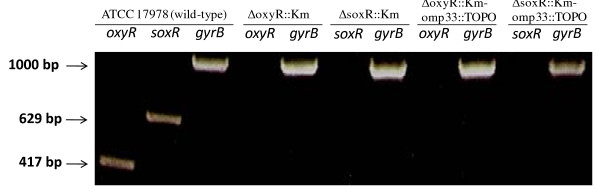
**Transcriptional analysis**. RT-PCR analysis of RNA extracted from the wild-type, ΔoxyR::Km, ΔsoxR::Km, ΔoxyR::Km-omp33::TOPO, and ΔsoxR::Km-omp33::TOPO strains showing the lack of *oxyR *and *soxR *transcription in the corresponding mutants. The *gyrB *gene was used as a housekeeping gene. The lengths of cDNAs obtained are indicated.

To ensure reproducibility of the gene replacement method, we produced the gene replacements of *oxyR *and *soxR *(Table 1). The same gene replacement method used to produce the Δomp33::Km mutant was also used to construct the ΔoxyR::Km and ΔsoxR::Km mutants (Figure 4), with the primers listed in Table 2. The PCR tests with locus-specific primers revealed that 2 of the 7 clones obtained for the *oxyR *gene, and all clones (3) obtained for the *soxR *gene had replaced the wild-type gene with the kanamycin resistance cassette (Figure 4). In addition, allelic replacement in mutant clones was further confirmed by sequencing the PCR products obtained (data not shown). Transcriptional analyses demonstrated the lack of both *oxyR *and *soxR *gene expression in the ΔoxyR::Km and ΔsoxR::Km mutants, respectively (Figure 5).

**Table 2:** Oligonucleotides used in the present study

Oligonucleotide name	Sequence (5' to 3')	Application
33intUP	CTGGTGACGTTGCTGGTACA	Construction of the omp33::TOPO mutant
33intDW	CGTTACCGATGATACCGAAG	Construction of the omp33::TOPO mutant
33extUP	CCTTAACATTACGTTTCATC	Confirmation of the omp33::TOPO mutant
33extDW	CATGTAAGATGCACCAACTGC	Confirmation of the omp33::TOPO mutant
Kmup	CCGGAATTGCCAGCTGGG	Kanamycin amplification
Kmdw	TTCAGAAGAACTCGTCAAG	Kanamycin amplification
33upFW	GCTGAGCTCGTAAAGTCTGATG	Construction and confirmation of the Δomp33::Km mutant
33upintRV	CCCAGCTGGCAATTCCGGGGCTAATAATACAGCAGTGG	Construction of the Δomp33::Km mutant
33dwintFW	CTTGACGAGTTCTTCTGAAGGCTTAAATGCTAAATTCCG	Construction of the Δomp33::Km mutant
33dwRV	CGTTGCCTTTTACCGTAGTC	Construction and confirmation of the Δomp33::Km mutant
33FWnest	GCAATTGAATTGTGTGAC	Construction of the Δomp33::Km mutant
33RVnest	TGATAGCAATTCAAGAGG	Construction of the Δomp33::Km mutant
OxyupFW	AGTTAAAAAAAATTGAAGAAA	Construction and confirmation of the ΔoxyR::Km mutant
OxyupintRV	CCCAGCTGGCAATTCCGGTCACTTGATGATCTCGATTTA	Construction of the ΔoxyR::Km mutant
OxydwintFW	CTTGACGAGTTCTTCTGAATGAAGATCACCAGTTAATGG	Construction of the ΔoxyR::Km mutant
OxydwRV	TATATTAACCATATTGAAGCC	Construction and confirmation of the ΔoxyR::Km mutant
OxyFWnest	GCAACTTGATGCAGCGGT	Construction of the ΔoxyR::Km mutant
OxyRVnest	TCAACGTAGCTACTATCC	Construction of the ΔoxyR::Km mutant
SoxupFW	ATGAAAGAAAAAAACTATATA	Construction and confirmation of the ΔsoxS::Km mutant
SoxupintRV	CCCAGCTGGCAATTCCGGATATTTACTTAGGGCTTGTTT	Construction of the ΔsoxS::Km mutant
SoxdwintFW	CTTGACGAGTTCTTCTGAAGAACAATGTCCATT AGAAA	Construction of the ΔsoxS::Km mutant
SoxdwRV	TCTGACTTCGTTTTTTGCTTA	Construction and confirmation of the ΔsoxS::Km mutant
SoxFWnest	AATTGCACGTTGCGATAG	Construction of the ΔsoxS::Km mutant
SoxRVnest	TAAACCAGATAGCCCAAC	Construction of the ΔsoxS::Km mutant
ATG33XbaI*	ATCGTCTAGACCCAGCTTTATCTCTTGTTA	Cloning of the *omp33 *gene
STOP33NcoI*	ATCGCCATGGGGACTGGACTCAGGAAGATTTG	Cloning of the *omp33 *gene
SoxRTup	AGGAACTGTAATTGCACG	RT-PCR for *soxR *detection
SoxRTrv	CCAATCGAGAGATAGCTC	RT-PCR for *soxR *detection
OxyRTup	ATGGCTGCATTACCCTCACT	RT-PCR for *oxyR *detection
OxyRTrv	GCGCTCTACAATCTTCTCAC	RT-PCR for *oxyR *detection
GyrBup	ATGAGTTCAGAGTCTCAATCA	RT-PCR for *gyrB *detection
GyrBrv	CTGTTAAACCTTCACGCGCAT	RT-PCR for *gyrB *detection
T7	AATACGACTCACTATAGGG	Universal primer of the pCR-BluntII-TOPO plasmid
SP6	ATTTAGGTGACACTATAG	Universal primer of the pCR-BluntII-TOPO plasmid
pETRAFW	TTCTTCGTGAAATAGTGATGATTTTT	Primer of the pET-RA plasmid
pETRARV	CTGTTTCATATGATCTGGGTATC	Primer of the pET-RA plasmid

*Oligonucleotides including the indicated restriction site (underlined)

### Construction of double knockout mutants

With the purpose of generating double knockout mutants, the recombinant plasmid pTOPO33int was transformed into both ΔoxyR::Km and ΔsoxR::Km mutants. After selection on zeocin- and kanamycin-containing plates, the ΔoxyR::Km-omp33::TOPO and ΔsoxR::Km-omp33::TOPO *A. baumannii *double knockout mutants were obtained. PCR tests with locus-specific primers revealed that both mutants had fragments of the expected size (data not shown). In addition, gene disruption in mutant clones was further confirmed by sequencing the PCR products obtained, by transcriptional analyses to detect the *oxyR *and *soxR *genes (Figure 5), and by Western blot analyses to detect the *omp33 *gene (data not shown).

## Discussion

Allelic mutation experiments enable investigation of the functions of many unknown genes identified during the sequencing of entire genomes. A number of methods can be used to inactivate bacterial chromosomal genes. As mentioned above, disruption of the *A. baumannii *chromosome can be achieved by integration of a plasmid into the chromosome by single crossover recombination [10]. For this purpose, an internal fragment that is homologous to the target gene must be cloned into a non-replicating plasmid carrying at least one antibiotic resistance cassette. However, the stability of this type of mutant must be taken into account, because if the gene-disrupted mutant cells are grown in a medium lacking antibiotic pressure, the integrated sequence could be removed, and the disrupted gene could revert to the original wild-type [16]. We tested this possibility, and found that this is indeed the case, as also found in similar studies with *E. coli *[16]. Therefore, one limitation of the method is that the resulting mutants should always be maintained in an appropriate medium containing selective antibiotics. Another disadvantage of the method is that further manipulations of the mutant strain are restricted, because the same vector cannot be used (because undesired recombination events would be highly likely), thus making it impossible to construct multiple gene knockout mutants.

The gene replacement method has recently been used to generate stable *A. baumannii *mutants [11-13]. This method is based on integration of a plasmid containing the inactivated gene of interest into the bacterial chromosome by single crossover recombination, followed by resolution (or excision) of the integrated DNA by a second recombination event, resulting in replacement of the original wild-type gene by the inactivated gene. The key step in this procedure, in *A. baumannii*, is counter-selection (widely used with Gram-negative bacteria) by the detection of sucrose sensitivity in the presence of the *Bacillus subtilis sacB *gene coding for levansucrase [17]. This method of counter-selection has been found to be useful for several other environmental bacteria [11,12,18]. Plasmids pSSK10, pEX100T, and pJQ200 have been successfully used to obtain *A. baumannii *mutants by this method [11-13]. However, most bacteria subjected to homologous recombination, even under negative selection for the *sacB *gene, are wild-type and it is not possible to isolate the desired mutant directly [19-21]. Another disadvantage of this method is that integration of the DNA may not always provide the desired replacement, since foreign DNA with low or no sequence homology would rely on illegitimate recombination events, as previously reported for *Acinetobacter *and other species [14,16]. In addition, all of these gene replacement methodologies are time-consuming, and require several steps involving subcloning into a suicide delivery vector followed by electroporation into *E. coli *and subsequent transfer into *A. baumannii *by electroporation or conjugation. To avoid such situations, we propose a method based on the electroporation of *A. baumannii *electrocompetent cells with linear DNA, a PCR product including an antibiotic resistance cassette flanked by regions homologous to the target locus. However, as expected, we noted an important disadvantage of the replacement method (which requires two recombination events), with respect to the gene disruption method (which only requires one recombination event), i.e. the low efficiency with regard to obtaining mutants (10^-7 ^vs. 10^-5^). In addition, we observed more illegitimate recombination events with the new method than with the gene disruption technique, since several colonies acquired the resistance antibiotic cassette (confirmed by PCR), although the wild-type target gene was not replaced (Figures 1, 2, and 4). Nevertheless, the new method is a useful genetic tool for systematic generation of knockouts. Moreover, to our knowledge, there are no previous reports of double knockout mutant strains of *A. baumannii*. However, we demonstrate that the combination of both gene disruption and gene replacement techniques is an easy and useful procedure for obtaining double gene-knockout mutants in *A. baumannii*.

Taking into account the results presented here, it intuitively appears that the gene replacement method would be successful with any strain of *A. baumannii*, including clinical strains, with the only limitation being the use of an appropriate antibiotic resistance marker. Although the kanamycin resistance cassette cannot be used in clinical strains (all the clinical strains of *A. baumannii *taken from our collection were kanamycin resistant: data not shown), use of another antibiotic resistance marker such as rifampicin (for which a low level of resistance has been demonstrated in approximately 50% of multidrug-resistant *A. baumannii *clinical isolates in Spain [22,23]), would be appropriate for generating mutants by gene replacement in these problematic strains.

## Conclusions

We report mutagenesis of three *A. baumannii *genes by use of a simple and rapid method. The method offers advantages such as no cloning steps, stability even in the absence of selective pressure, and the possibility of constructing multiple gene knockout mutants. The method may therefore facilitate the understanding of the genetics of *A. baumannii*. Although not tested, it is also possible that this novel method may also work with other pathogenic bacteria, in which genetic manipulation techniques are generally less well established than for *E. coli *and other bacterial species. Finally, the gene disruption method is recommended when only one *A. baumannii *gene must be inactivated, and when it is possible to maintain selective pressure, since it is the fastest and most efficient method of producing *A. baumannii *mutants described so far.

## Methods

### Bacterial strains, plasmids, and growth conditions

Bacterial strains and plasmids used in this study are listed in Table 3. The *E. coli *and *A. baumannii *strains were grown in Luria Bertani (LB) medium [24]. When necessary, kanamycin (50 μg/ml), rifampicin (50 μg/ml), and zeocin (20 μg/ml for *E. coli *and 200 μg/ml for *A. baumannii*) were added to the growth media. All cultures were incubated at 37°C, 180 rpm. The frequency of generation of mutants was calculated as the number of mutants obtained, divided by the total CFU.

**Table 3:** Bacterial strains and plasmids used in the present study

Strain or plasmid	Relevant feature(s)	Source or reference
**Strains**		
*Acinetobacter baumannii*		
ATCC 17978	Wild-type strain	Laboratory stock
omp33::TOPO	Derived from ATCC 17978. *omp33 *mutant obtained by plasmid insertion. Kan^R^, Zeo^R^	Present study
Δomp33::Km	Derived from ATCC 17978. *omp33 *mutant obtained by gene replacement. Kan^R^	Present study
ΔoxyR::Km	Derived from ATCC 17978. *oxyR *mutant obtained by gene replacement. Kan^R^	Present study
ΔsoxR::Km	Derived from ATCC 17978. *soxR *mutant obtained by gene replacement. Kan^R^	Present study
ΔoxyR::Km-omp33::TOPO	Derived from ΔoxyR::Km. *oxyR omp33 *double mutant. Kan^R^, Zeo^R^	Present study
ΔsoxR::Km-omp33::TOPO	Derived from ΔsoxR::Km. *soxR omp33 *double mutant. Kan^R^, Zeo^R^	Present study
*Escherichia coli*		
TG1	supE thi-1 Δ(*lac*-*pro*AB) Δ(*mcr*B-*hsd*SM)5(rK- mK-) [F' *traD*36 *pro*AB *lac*IqZΔ*M15*]	Laboratory stock
**Plasmids**		
pCR-BluntII-TOPO	Suicide plasmid for *A. baumannii*. Kan^R^, Zeo^R^	Invitrogen
pTOPO33int	pCR-BluntII-TOPO containing a 387-pb internal fragment of the *omp33 *gene. Kan^R^, Zeo^R^	Present study
pET-RA	*A. baumannii *replication origin. CTX-M14 β-lactamase gene promoter. Rif^R^	Present study
pET-RA-OMP33	pET-RA containing the *omp33 *gene without its promoter region. Rif^R^	Present study

Kan, kanamycin; Zeo, zeocin; Rif, rifampicin

### DNA manipulations

Genomic and plasmid isolation were performed with a Wizard Genomic DNA Purification Kit (Promega) and Wizard Plus SV Minipreps DNA Purification System (Promega), respectively. Restriction enzymes and DNA-modifying enzymes were purchased from Promega and used according to the manufacturer's recommendations. Standard PCR amplifications were performed with BioTaq DNA polymerase (Bioline). When necessary, high fidelity and blunt-ended PCR products were amplified with Expand High Fidelity (Roche) and Accuzyme (Bioline) DNA polymerases, respectively. All oligonucleotides (Sigma) used in the study are listed in Table 2. PCR products were purified with the High Pure PCR Product Purification Kit (Roche). When high concentrations of purified PCR products were required, a MinElute PCR Purification Kit (Qiagen) was used. All the recombinant plasmids obtained in the study, and the PCR products indicated, were sequenced by the Macrogen sequencing service (Seoul, Korea).

### Electroporation

All strains were made electrocompetent as follows. Bacterial overnight cultures were grown in LB broth and subcultured at a dilution of 1:20 in 100 ml of fresh LB medium. Cultures were grown at an OD_600 _of 0.8 and then incubated on ice for 10 min. Cells were pelleted by centrifugation and then washed 3 times with 10% (v/v) glycerol and finally resuspended in 500 μl of 10% (v/v) glycerol. An aliquot of 100 μl of the cell suspension was mixed with the recombinant DNA (up to 20 μl). The mixture was placed in a pre-chilled sterile electroporation cuvette (1 mm electrode gap, Bio-Rad) and immediately pulsed by use of a Bio-Rad Gene Pulser (1.8 kV, 200 W, and 25 μF). The mixture was incubated at 37°C for 1 h with 1 ml of LB broth. Cells were spread on LB agar containing the appropriate antibiotics and incubated at 37°C.

### Knockout construction by gene replacement

**Figure 6:**
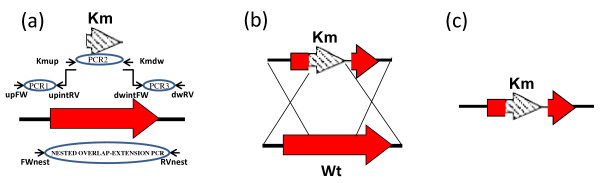
**Gene replacement**. (a) Schematic representation of the strategy used to construct mutants by gene replacement. Small, red and shaded arrows represent the primers, the target gene, and the kanamycin (Km) resistance cassette, respectively. The three PCR products obtained (PCR1, PCR2, and PCR3) were mixed at equimolar concentrations and subjected to a nested overlap-extension PCR to generate the desired linear DNA (see Materials and Methods for details). (b) Diagram showing the integration of the linear DNA via two recombination events. (c) Representation of the original genetic material replaced by the recombinant DNA on the *A. baumannii *chromosome.

The upstream and downstream regions (approximately 0.5 kbp each) of the target gene were amplified from genomic DNA of *A. baumannii *ATCC 17978 strain using primer pairs upFW + upintRV and dwintFW + dwRV (Figure 6), respectively. The kanamycin cassette was amplified using primers Kmup and Kmdw (Table 2) and the pCR-BluntII-TOPO vector (Invitrogen) as a template. The upintRV and dwintFW primers (Figure 6) contained, at their 5' ends, an extension of approximately 20 nucleotides homologous to the Kmup and Kmdw primers, respectively. The three PCR products obtained in the first step were mixed at equimolar concentrations and subjected to a nested overlap-extension PCR with FWnest and RVnest primers (Figure 6) to generate a kanamycin resistance cassette flanked by both the upstream and the downstream gene homologous regions. The nested overlap-extension PCR was carried out with an Expand High Fidelity Taq DNA polymerase (Roche), according to the manufacturer's recommendations; the conditions used were as follows: 94°C for 15 s, 40°C for 1 min, 72°C for 2 min (10 cycles); 94°C for 15 s, 55°C for 1 min, 72°C for 3 min (20 cycles), and a final extension at 68°C for 10 min. Electroporation of the *A. baumannii *ATCC 17978 strain was performed with approximately 5 μg of the purified PCR product containing the inactivated gene. Cells were then plated on LB agar containing kanamycin for selection of mutants whose wild-type genes were replaced by allelic exchange via double crossover recombination. Gene replacement in candidate clones was verified by PCR with upFW and dwRV primers (Figure 6). Allelic replacement in candidate clones was further confirmed by sequencing the mutant region in the resulting mutants.

### Knockout construction by gene disruption

Plasmid insertion in the *omp33 *gene (Table 1) was carried out as previously described [10], with slight modifications. Briefly, kanamycin- and zeocin-resistant plasmid pCR-BluntII-TOPO, unable to replicate in *A. baumannii*, was used as a suicide vector. An internal fragment (387 bp) of the *omp33 *gene was amplified by PCR with 33intUP and 33intDW primers (Table 2) and genomic DNA from *A. baumannii *ATCC 17978 as a template. The PCR product was cloned into the pCR-BluntII-TOPO vector and electroporated in *E. coli *to yield the pTOPO33int plasmid (Table 3). Recombinant plasmid (0.1 μg) was then introduced in the kanamycin- and zeocin-susceptible *A. baumannii *ATCC 17978 strain by electroporation. Mutants were selected on kanamycin-containing plates. Inactivation of the *omp33 *gene by insertion of the plasmid via single crossover recombination was confirmed by sequencing the amplified PCR products with the SP6 + 33extUP and T7 + 33extDW primer pairs (Table 2).

### Construction of pET-RA plasmid for gene expression in *A. baumannii*

**Figure 7:**
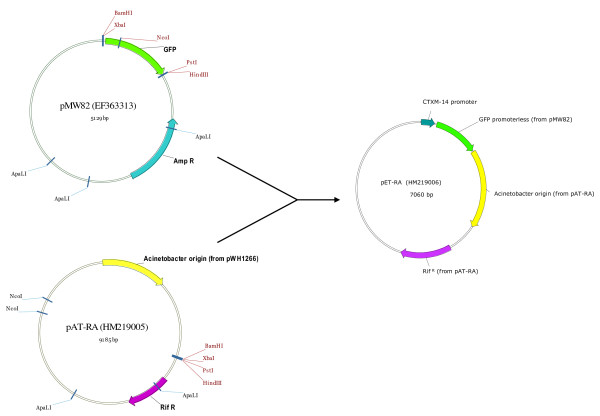
**pET-RA construction**. Schematic representation of the construction of the pET-RA plasmid. The GenBank accession numbers of the plasmids are indicated in parenthesis. Rif, rifampicin; Amp, ampicillin; GFP, green fluorescent protein.

In order to complement mutant phenotypes, the pET-RA plasmid [Genbank: HM219006] was constructed, and carried a rifampicin resistance cassette, a gene coding for a green fluorescent protein (GFP), and the *A. baumannii *replication origin, which is a plasmid origin of replication (Figure 7). The pET-RA vector was used to express promoterless genes under control of the CTX-M14 β-lactamase gene promoter, previously cloned upstream of the GFP gene (Figure 7). For pET-RA construction, the pMW82 vector [Genbank: EF363313] was amplified by PCR, excluding the coding region of the ampicillin resistance cassette. The rifampicin resistance cassette was then amplified from the pAT-RA vector [Genbank: HM219005] and introduced into the pMW82 vector. The CTXM-14 promoter was cloned into the pMW82 vector *BamH*I site. The *Acinetobacter *replication origin was amplified from the pAT-RA vector and cloned into the *Hind*III site of the pMW82 vector. As a result, the pET-RA vector containing the CTXM-14 promoter was obtained and used for cloning and expression of genes in the *Xba*I-*Nco*I restriction sites.

### Complementation of *omp33 *mutants

In order to complement the *A. baumannii omp33 *mutants, the omp33 ORF was amplified with the ATG33XbaI and STOP33NcoI primers (Table 2) from the *A. baumannii *ATCC 17978 strain genome and cloned into the *Xba*I-*Nco*I restriction sites of the pET-RA vector under the control of the β-lactamase CXT-M-14 gene promoter yielding the pET-RA-OMP33 plasmid (Table 3). *Acinetobacter baumannii omp33 *mutants were transformed with the recombinant pETRA-OMP33 plasmid. Transformants were selected on rifampicin- and kanamycin-containing plates and confirmed by PCR with the pETRAFW and pETRARV primers (Table 2).

### Mutant stability assays

The bacterial cultures were grown in 5 ml of LB broth without kanamycin and incubated at 37°C. Every day, during 10 consecutive days, 100 μl of each culture was diluted in 5 ml of fresh medium and incubated for 24 h. The same experiment was also carried out, for each strain, with medium containing kanamycin. On days 1, 5, and 10, all cultures were diluted 10^6^-fold and dilutions (0.1 ml) were plated on non-selective plates. From these plates, 100 colonies were each transferred to non-selective and selective plates to determine the frequency of revertants, on the basis of the percentage of kanamycin-susceptible colonies.

### RNA methods

Bacterial cultures were grown overnight in LB broth supplemented with the appropriate antibiotics at 37°C. RNA isolation was performed with the RNAeasy Plant Mini Kit (Qiagen). The total RNA extraction was subjected to DNaseI (Invitrogen) treatment, following the manufacturer's instructions. In order to evaluate transcription of the genes of interest, an RT-PCR was performed with the First Strand cDNA Transcriptor Synthesis Kit (Roche) and the *gyrB *as housekeeping gene. Both the first strand synthesis and the PCR amplification were carried out with the specific primers listed in Table 2. Finally, RT-PCR products were visualized in a 1% agarose gel.

### Protein analysis

Extraction of *A. baumannii *cell surface-associated proteins and two-dimensional gel electrophoresis (2-DE) were performed as described elsewhere [15]. Proteins were quantified by the Bradford assay, as previously described [25]. Forty μg of protein from each sample was loaded onto a sodium dodecyl sulphate-polyacrylamide gel (12%) in a minigel apparatus (Bio-Rad) and transferred to a Polyvinylidene Fluoride (PVDF) membrane (Roche). To ensure transfer of proteins from the gel, the membrane was stained with Ponceau red. The membrane was then washed, blocked with 5% (wt/vol) blocking agent (non-fat skimmed milk), and incubated with a primary antibody against Omp33 obtained from mouse (INIBIC, A Coruña). Proteins were visualized by incubation with horseradish peroxidase-conjugated secondary antibody, followed by enhanced chemiluminescence ECL Plus (Amersham Pharmacia Biotech) and detected with the LAS3000 chemiluminescence detector (Fujifilm).

## Authors' contributions

JA and MP conceived the design of the study, carried out several experimental procedures, and drafted the manuscript. BG and SR participated in the mutant construction and complementation. CR and JR carried out the protein analysis. PR carried out the construction of pET-RA plasmid. GB participated in the design and coordination of the study and helped to draft the manuscript. All authors read and approved the final manuscript.
